# Ca^2+^/calmodulin regulates Kvβ1.1-mediated inactivation of voltage-gated K^+^ channels

**DOI:** 10.1038/srep15509

**Published:** 2015-10-21

**Authors:** Sandip M. Swain, Nirakar Sahoo, Sophie Dennhardt, Roland Schönherr, Stefan H. Heinemann

**Affiliations:** 1 Center for Molecular Biomedicine, Department of Biophysics, Friedrich Schiller University Jena & Jena University Hospital, Hans-Knöll-Str. 2, D-07745 Jena, Germany

## Abstract

A-type K^+^ channels open on membrane depolarization and undergo subsequent rapid inactivation such that they are ideally suited for fine-tuning the electrical signaling in neurons and muscle cells. Channel inactivation mostly follows the so-called ball-and-chain mechanism, in which the N-terminal structures of either the K^+^ channel’s α or β subunits occlude the channel pore entry facing the cytosol. Inactivation of Kv1.1 and Kv1.4 channels induced by Kvβ1.1 subunits is profoundly decelerated in response to a rise in the intracellular Ca^2+^ concentration, thus making the affected channel complexes negative feedback regulators to limit neuronal overexcitation. With electrophysiological and biochemical experiments we show that the Ca^2+^ dependence is gained by binding of calmodulin to the “chain” segment of Kvβ1.1 thereby compromising the mobility of the inactivation particle. Furthermore, inactivation regulation via Ca^2+^/calmodulin does not interfere with the β subunit’s enzymatic activity as an NADPH-dependent oxidoreductase, thus rendering the Kvβ1.1 subunit a multifunctional receptor that integrates cytosolic signals to be transduced to altered electrical cellular activity.

The activity of voltage-dependent K^+^ channels (Kv channels) typically counteracts electrical excitation of cells, such as neurons or muscle cells. To precisely match the need of such counteraction with the shape and frequency of action potentials, some K^+^ channels, often referred to as A-type channels, undergo rapid voltage-dependent inactivation; they are responsible for regulating the action potential width and afterhyperpolarization, and thus Ca^2+^ entry and neurotransmitter release^1^,^2^. The molecular mechanism for rapidly inactivating A-type channels mostly is of the “ball-and-chain” type: the K^+^-selective pore of the channels, which are formed of four α subunits with each six transmembrane segments and cytosolic N and C termini, is occluded by one of the four cytosolic N termini that block the internal mouth of the channel^3^. Inactivation is abolished by deletion of the N termini, and it can be restored by the intracellular application of peptides derived from such N-type inactivating channels^4^,^5^. The N terminus itself is an intrinsically disordered part of the protein, divided into a distal “ball” segment that actually obstructs the pore, and a flexible “chain” that provides mobility and determines the kinetics of inactivation. Among mammalian K^+^ channel α subunits, only a few are capable of inducing N-type inactivation (e.g., Kv1.4, Kv3.4)^6^. However, auxiliary cytoplasmic Kvβ subunits with a similar N-terminal “ball” domain may also interact with Kv1 family α subunits to create N(β)-type inactivating A-type channels^7^,^8^,^9^. Kvβ subunits consist of a highly conserved core domain with structural homology to NAD(P)H-dependent oxidoreductases^10^ that bind to the N termini of Kvα subunits^11^,^12^, and diverse N-terminal structures, some of which serving as “ball-and-chain” inactivation domains^9^.

Such Kvβ subunits capable of transforming non-inactivating delayed rectifier K^+^ channels into inactivating A-type channels not only expand the diversity of K^+^ current inactivation kinetics found *in vivo*, they also substantially contribute to various means of regulation by which cells fine-tune K^+^ channel inactivation and thereby regulate electrical excitation. For example, cysteine residues in the “ball” domain make N(β)-type inactivation sensitive to changes in the intracellular redox milieu as with Kv1.4 and Kv3.4 channels^6^,^7^,^13^,^14^. Furthermore, the degree and kinetics of N(β) inactivation depend on the state of phosphorylation^15^,^16^,^17^, lipid composition^18^ and the intracellular pH^19^. The enzymatic activity of the Kvβ’s core domains to act as oxidoreductases^20^ also has an impact on N(β) inactivation because the mobility of the “chain” depends on whether the subunit is complexed with NADPH or NADP^+^, i.e. enzymatic activity and electrophysiological function are coupled^21^,^22^.

Most importantly, an increase in the intracellular Ca^2+^ concentration slows down inactivation induced by Kvβ1.1 subunits, and the N terminus of these subunits appeared to be necessary for that function^23^. Thus, K^+^ channels that undergo inactivation by means of Kvβ1.1 subunits provide a negative feedback regulation, as they tend to limit excitation in response to Ca^2+^ overload. This phenomenon is not universal because inactivation induced by the N-terminal splice variant Kvβ1.3 does not depend on [Ca^2+^]_i_ 24. However, the molecular mechanism underlying the Ca^2+^ dependence of Kvβ1.1-mediated inactivation remained to be elucidated. Knowledge of this mechanism would furthermore allow addressing the question of a potential crosstalk between Ca^2+^ sensitivity on the one hand and dependence of Kvβ-induced inactivation on the enzymatic activity on the other.

Here, we identified calmodulin (CaM) as the Ca^2+^ sensor protein responsible for the Ca^2+^ sensitivity of Kvβ1.1–induced K^+^ channel inactivation. This Ca^2+^/CaM dependent inactivation modulation was independent of cellular oxidation and of the intrinsic enzymatic activity of Kvβ1.1.

## Results

### Ca^2+^ dependence of Kvβ-induced inactivation

**Figure 1:**
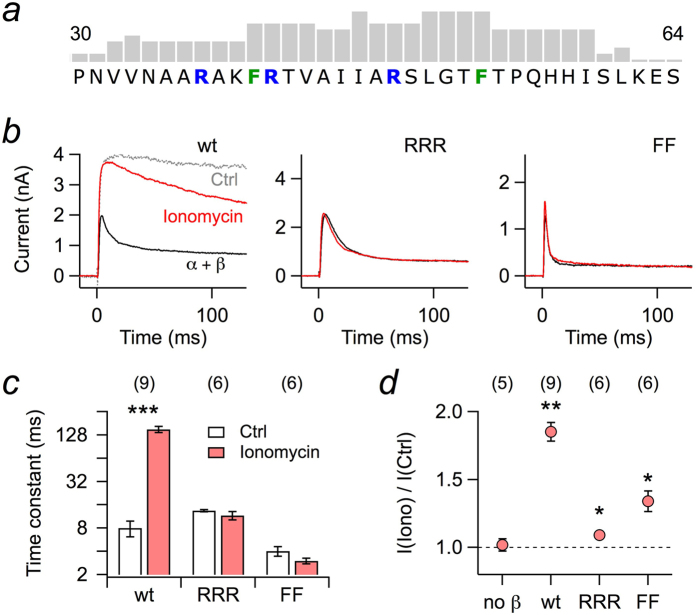
Whole-cell recordings of Kv1.1 currents from HEK 293T cells. (**a**) Part of the N-terminal sequence of rat Kvβ1.1 protein with score pattern resulting from a search for potential calmodulin binding sites according to Mruk *et al.* (2014)^25^; the tallest bar refers to a score value of 9. Within this motif, either the marked arginine residues (RRR) or both phenylalanines (FF) were mutated to asparagine and serine, respectively. (**b**) Kv1.1 channels were coexpressed with Kvβ1.1 wild type (wt) or mutants RRR and FF in HEK 293T cells; currents were measured upon depolarization to 50 mV. The pipette solution contained 100 μM EGTA. Current traces for the indicated Kvβ1.1 subunits before (black) and after (red) extracellular application of 1 μM ionomycin. The grey trace in the left panel (Ctrl) indicates Kv1.1 currents without Kvβ subunits. (**c**) Inactivation time constants, based on single-exponential fits from data as shown in panel b. (**d**) Fractional change in peak current at 50 mV upon ionomycin application. Data in c and d are mean ± s.e.m. with n indicated in parentheses. Two-sided paired t-test between control and ionomycin application in c, Wilcoxon signed rank test in d: ***P < 0.001, **P < 0.01, *P < 0.05.

It was previously shown that rapid N-type inactivation of Kv1.1 channels when coexpressed with Kvβ1.1 depends on the level of free intracellular Ca^2+^ 23,24. This phenomenon is readily observed when Kv1.1 and Kvβ1.1 are coexpressed in HEK 293T cells and currents are recorded in the whole-cell patch-clamp mode with an intracellular solution only weakly Ca^2+^-buffered with 100 μM EGTA. Under this condition, Kvβ1.1 induced rapid inactivation when channels were activated by a depolarizing step (Fig. 1b, black), characterized by an inactivation time constant of 8.01 ± 1.85 ms. Upon extracellular application of the Ca^2+^ ionophore ionomycin, however, the inactivation was slowed down considerably (151 ± 13.5 ms) and the peak outward current was increased about twofold (Fig. 1b,c, red). No change in peak current upon ionomycin application was observed when Kv1.1 was expressed alone.

In search for the molecular mechanism underlying this regulation, we analyzed the N-terminal sequence of Kvβ1.1 and also of other Kvβ subunits for potential interaction motifs for Ca^2+^-binding proteins, such as calmodulin (CaM). While the “Calmodulation database and meta-analysis predictor” algorithm according to Mruk *et al.* (2014)^25^ did not predict CaM binding sites in the N-terminal sequences of Kvβ1.2, Kvβ1.3, and Kvβ3.1, i.e. Kvβ subunits that can induce N-type inactivation^9^, there was a high score in the N-terminal sequence of Kvβ1.1, ranging from position F40 through I59 (Fig. 1a), i.e. directly following the N-terminal sequence that presumably forms the “ball” domain (extending to residue 34). In this region, there are typical features of a CaM-binding structure comprising basic residues (R37, R41, R48) and aromatic residues (F40, F53). To elucidate if such structures are involved in the observed Ca^2+^ dependence of Kvβ1.1-induced inactivation, asparagine was replaced for the arginines yielding the Kvβ1.1 mutant RRR and serine for phenylalanines to yield the mutant Kvβ1.1-FF. Upon coexpression with Kv1.1 in HEK 293T cells, both constructs induced rapid inactivation; inactivation by RRR was slightly slower than that of the wild type (13.5 ± 0.4 ms), while inactivation induced by FF was about 2.5-fold faster (4.0 ± 0.6 ms) (Fig. 1b,c). Application of ionomycin, however, did not significantly affect the time course of inactivation in either case (Fig. 1c). The peak currents obtained for the coexpression of RRR were only increased by 9.9 ± 2.4%, that for FF by 34 ± 7.6% (Fig. 1d).

These data clearly indicate that the putative CaM-binding motif is involved in the Ca^2+^ dependence of Kvβ1.1-induced inactivation. The impact of the mutations on inactivation was also observed when currents were recorded in the whole-cell mode with defined intracellular solutions containing either no free Ca^2+^ (buffered with 10 mM EGTA) or 1 μM Ca^2+^ (Supplementary Fig. S1a–c). Furthermore, intracellular Ca^2+^ did not affect the time course of recovery from inactivation, indicating that the offrate of N(β)-terminal inactivation is not Ca^2+^ dependent either (Supplementary Fig. S1d).

**Figure 2:**
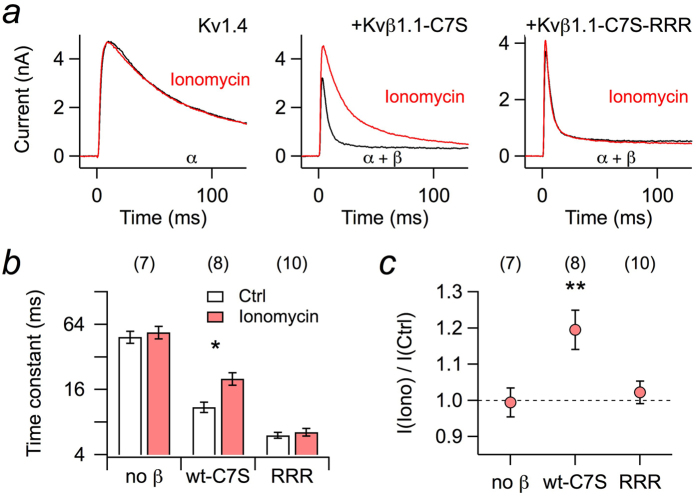
Whole-cell recordings of Kv1.4 current from HEK 293T cells. (**a**) Kv1.4 channels were expressed in HEK 293T cells alone (*left*) or in combination with Kvβ1.1-C7S (center) or mutant Kvβ1.1-C7S-RRR (right); currents were measured upon depolarization to 50 mV before (black) and after (red) extracellular application of 1 μM ionomycin. The pipette solution contained 100 μM EGTA. (**b**) Inactivation time constants, based on single-exponential fits from data as shown in panel a. (**c**) Fractional change in peak current at 50 mV upon ionomycin application. Data in b and c are mean ± s.e.m. with n indicated in parentheses. Two-sided paired t-test between control and ionomycin application in b, Wilcoxon signed rank test in c: **P < 0.01; *P < 0.05.

Within the Kv1 subfamily of Kv channels, Kv1.4 α subunits harbor N-terminal domains that also induce channel inactivation. When expressed in HEK 293T cells and subjected to the application of ionomycin to elevate the intracellular Ca^2+^ concentration, the kinetics of inactivation, however, was not altered (Fig. 2a, *left*), thus rendering the N-type inactivation endogenous to Kv1.4 channels insensitive to acute increases in [Ca^2+^]_i_. However, in a physiological setting Kv1.4 often coassembles with Kvβ1.1 subunits, thus yielding Kv channel complexes with eight N-terminal inactivation domains^26^. As a result, inactivation at 50 mV, which is approximated with a single-exponential time constant of 49.1 ± 6.3 ms (n = 7) for Kv1.4, is substantially accelerated in the presence of Kvβ1.1-C7S (11.0 ± 1.2 ms, n = 8). We used mutant Kvβ1.1-C7S to avoid any confounding with the redox milieu and a potential crosstalk with the cysteine (C13) in the “ball” domain of Kv1.4. Application of ionomycin slowed down this inactivation about twofold (*P *< 0.05; Fig. 2a, *center*, Fig. 2b). Coexpression of mutant Kvβ1.1-C7S-RRR made the inactivation of the Kv1.4/Kvβ1.1-C7S-RRR complex even faster (6.05 ± 0.38 ms, n = 10), but ionomycin was without effect (*P *= 0.12; Fig. 2a, *right*, Fig. 2b). In addition to a deceleration of the inactivation, elevated [Ca^2+^]_i_ also increased the peak outward current in the combination of Kv1.4/Kvβ1.1-C7S but not with Kv1.4 alone or when coexpressed with Kvβ1.1-C7S-RRR (Fig. 2c). Thus, by means of coassembly with Kvβ1.1 subunits, Kv1.4 channels not only acquire a faster N-type inactivation but also one that is regulated by intracellular Ca^2+^.

### Calmodulin mediates Ca^2+^ sensitivity of Kvβ1.1

**Figure 3:**
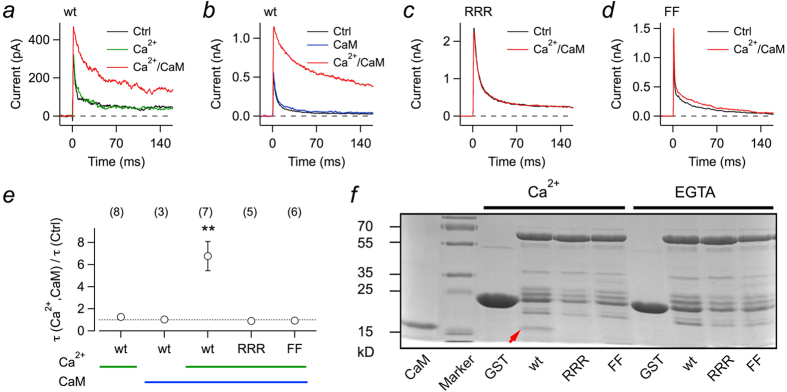
Calmodulin (CaM) is required for Ca^2+^-dependent inactivation mediated by Kvβ1.1. (**a**–**d**) Inside-out patch recordings from *Xenopus* oocytes coexpressing Kv1.1 with Kvβ1.1 wild type (wt) (**a**,**b**) and mutants RRR (**c**) and FF (**d**). Current traces were obtained at 50 mV. The bath solution facing the intracellular side contained no Ca^2+^ and no CaM (Ctrl, black), 1 μM Ca^2+^ (green), 1 μM CaM (blue), or 1 μM Ca^2+^ plus 1 μM CaM (red). (**e**) Relative change in fast inactivation time constant for Kvβ1.1 wild type and the mutants for the indicated application of intracellular Ca^2+^ and CaM. Data are mean ±  s.e.m. with n indicated in parentheses. Two-sided paired t-test between control and ionomycin application: **P < 0.01. The intracellular solution contained 1 mM glutathione (reduced). (**f**) SDS PAGE of the GST-pull-down assay to test for binding of recombinant CaM to sepharose-bound GST alone (GST) or GST-fused Kvβ1.1 variants (wt, RRR, FF). Precipitation of CaM was only observed with GST-fused wild-type Kvβ1.1 in the presence of Ca^2+^ ions (arrow). GST fusions of the Kvβ1.1 mutants RRR or FF did not co-precipitate CaM. The lane labeled “CaM” shows recombinant CaM as a control.

To gain direct access to the cytosolic side of the plasma membrane with functional Kv1.1/Kvβ1.1 complexes, we expressed the corresponding mRNAs in *Xenopus* oocytes and obtained macro-patches in the inside-out configuration. In this mode, various solutions can be directly applied to assay the channels’ dependence on Ca^2+^ and calmodulin. When a membrane patch was excised into a solution devoid of Ca^2+^ and CaM, rapid inactivation was observed (Fig. 3a, black). Application of solutions with 1 μM free Ca^2+^ did not have any impact on the current (Fig. 3a, green); 1 μM Ca^2+^ substantially slowed down inactivation only when coapplied with 1 μM recombinantly produced CaM (Fig. 3a, red). A similar experiment is shown in Fig. 3b, illustrating that CaM in the absence of Ca^2+^ has no effect, and only Ca^2+^/CaM in combination remove inactivation induced by Kvβ1.1. For mutants RRR (Fig. 3c) and FF (Fig. 3d), however, even Ca^2+^/CaM was ineffective in removing inactivation as also illustrated in Fig. 3e as mean over multiple experiments.

GST-pull-down assays were performed to study the physical interaction of CaM with Kvβ1.1. Co-precipitation of CaM with GSH sepharose-bound proteins was only observed with GST-fused wild-type Kvβ1.1 in the presence of free Ca^2+^ ions. By contrast, GST fusions of the Kvβ1.1 mutants RRR or FF did not co-precipitate CaM in this binding assay (Fig. 3f). This finding suggests that only one CaM binding site exists in the N terminus of Kvβ1.1.

The lack of a Ca^2+^ effect on inactivation in the absence of CaM appears to be in contrast to the report of Jow *et al.* (2004)^23^ who showed a Ca^2+^ dependence of the Kvβ1.1-induced inactivation time constant in inside-out patches without CaM application. Although we cannot offer an unequivocal explanation for that observation, we noticed that the amount of cytosol that sticks to the membrane patch upon establishment of the inside-out configuration has a strong influence on how much endogenous CaM is available to facilitate loss of inactivation. As illustrated in Supplementary Fig. S2 for such patches with some cytosol adhering, the inactivation time course of Kv1.1+Kvβ1.1 complexes is fast in the on-cell configuration. Immediately upon patch excision into bath solution with 1 μM free Ca^2+^, however, channels do not inactivate anymore; yet inactivation is restored by transfer of the patch into Ca^2+^-free solution and subsequently inactivation is preserved even in 1 μM free Ca^2+^.

### Ca^2+^ sensitivity of Kvβ1.1 is functionally independent of its enzymatic activity

Kvβ subunits exhibit an enzymatic activity as aldoketoreductases using NADPH as a cofactor^20^. For Kvβ1.1 with its N-terminal structure leading to N(β)-type inactivation, enzymatic activity, e.g. induced by the application of the substrate 4-cyanobenzaldehyde (4CY), results in a significant slow-down of inactivation^21^. A mutagenesis study by Pan *et al.* (2011)^22^ provided an explanation for this phenomenon, in which an immobilization of the N-terminal ball domain is induced by oxidation of the cofactor NADPH, bound to the core domain of Kvβ1.1, to NADP^+^; in this state, the chain of the inactivation domain binds to the Kvβ1.1 core domain such that the distal ball domain cannot reach its receptor anymore and, hence, is unable to induce inactivation. Key players for the electrostatic coupling of “chain” and “core” domain are residues R37/R48 and E265/E349, respectively. Since the arginine residues R37 and R48 are apparently taking part in binding CaM to the Kvβ1.1 subunit, it is plausible that the regulation of Kvβ1.1-induced inactivation by NADPH/NADP^+^ and Ca^2+^/CaM are coupled.

**Figure 4:**
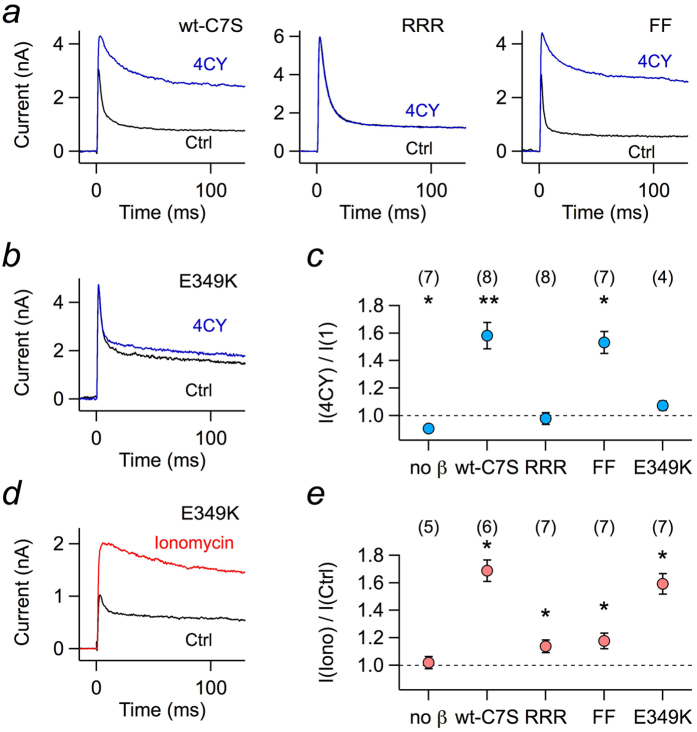
Enzymatic activity of Kvβ1.1-C7S variants. (**a**) Superposition of current traces at 50 mV of Kv1.1 coexpressed in HEK 293T cells with Kvβ1.1-C7S (wt-C7S) or its mutants RRR and FF right after establishment of the whole-cell configuration (black) and about 150 s thereafter (blue). The pipette solution contained 1 mM of the substrate 4CY. (**b**) As in a, but with mutant Kvβ1.1-C7S-E349K. (**c**) Relative change in peak current from experiments as in a and b for Kv1.1 (no β) and coexpression with the indicated variants of Kvβ1.1-C7S. (**d**) Superposition of current traces at 50 mV for Kv1.1 plus Kvβ1.1-C7S-E349K before (black) and after extracellular application of 1 μM ionomycin (red). (**e**) Fold change of peak current upon ionomycin application for Kv1.1 (no β) and coexpression with the indicated variants of Kvβ1.1-C7S. The pipette solution contained 100 μM EGTA. Data in c and e are mean ± s.e.m. with n indicated in parentheses. Deviation from unity was tested with a Wilcoxon signed rank test: **P < 0.01, *P < 0.05.

We therefore measured the impact of Kvβ1.1 enzymatic activity for the mutants that eliminated the Ca^2+^/CaM dependence. Since the enzymatic activity requires oxidizing conditions, which would eliminate inactivation of Kvβ1.1 by means of the regulatory N-terminal cysteine (C7)^7^, we used mutant Kvβ1.1-C7S and expressed it together with Kv1.1 in HEK 293T cells. Patch pipettes contained 1 mM of the substrate 4CY. Currents in response to depolarization to 50 mV were measured immediately after establishment of the whole-cell configuration, yielding inactivating K^+^ currents. During the course of substrate diffusing into the cytosol, the inactivation time course became progressively slower for Kvβ1.1-C7S (Fig. 4a, *left*). As expected, this did not happen for mutant RRR (Fig. 4a, *center*). However, 4CY also potently slowed down inactivation induced by Kvβ1.1-FF (Fig. 4a, *right*). As a control, we also measured mutant Kvβ1.1-E349K, which did not show a significant response to 4CY (Fig. 4b,c). Thus, as summarized in Fig. 4c, the substrate 4CY potently affected the inactivation time course of Kvβ1.1-C7S and Kvβ1.1-C7S-FF, but not of Kvβ1.1-C7S-RRR and Kvβ1.1-C7S-E349K, demonstrating that an intact Ca^2+^/CaM dependence is not required for the inactivation modulation via NADPH oxidation. Conversely, we measured the impact of elevated Ca^2+^ level on such constructs, as shown in Fig. 4d,e. Application of ionomycin to HEK 293T cells expressing Kv1.1+Kvβ1.1-C7S-E349K removed inactivation efficiently. In addition, the other mutations in the background of Kvβ1.1-C7S (Fig. 4e) showed the same dependence on [Ca^2+^]_i_ elevation as measured for Kvβ1.1 wild type (Fig. 1d), thus in summary illustrating that the removal of N(β)-type inactivation induced by Ca^2+^/CaM is not associated with a restraint of the chain flexibility that results from docking of R37/R48 in the chain to E265/E349 in the core domain of Kvβ1.1.

## Discussion

The auxiliary Kvβ1.1 subunit converts voltage-gated K^+^ channels formed of α subunits of the Kv1 subfamily to rapidly inactivating A-type channels when coexpressed in heterologous systems^7^,^8^. In the mammalian brain, Kvβ1.1 subunits coexpress with Kv1.1 and Kv1.4 in nerve terminals of cortical interneurons, mossy fibers, and in the substantia nigra^26^, where they regulate action potential frequency and shape, and thus also neurotransmitter release. Slow-down of A-type channel inactivation delays firing of action potentials and reduces the cell excitability by controlling the Ca^2+^ inflow^27^. The slow inactivation of Kv1.4 channels can produce progressive spike broadening and alteration of the action potential frequency^28^. The relevance of Kvβ-mediated inactivation and its precise tuning is underscored by the fact that various physiological parameters, such as the cellular redox status, cytosolic pH, phosphorylation signaling, and intracellular Ca^2+^ can modulate this process. It is now clear that Kvβ1.1 is able to combine and integrate signals from diverse pathways, coupling cellular excitability to cell physiology.

In the present study, we demonstrate that inactivation conferred to Kv1.1 and Kv1.4 channels by means of Kvβ1.1 subunits is considerably slowed down by intracellular Ca^2+^, and that this Ca^2+^ sensitivity arises from calmodulin, which binds to the “chain“ structure of Kvβ1.1 subunits. By means of this mechanism, K^+^ channels formed of Kv1.1/Kvβ1.1 or Kv1.4/Kvβ1.1 provide a negative feedback in the regulation of neuronal excitability and synaptic efficacy by coupling intracellular [Ca^2+^] to the hyperpolarizing activity of K^+^ outward flow and therefore potentially being important for various types of neurons. For example, fast N-type inactivation induced by Kvβ1.1 is linked to autosomal dominant lateral temporal lobe epilepsy^29^, such that Ca^2+^/CaM-mediated modulation of Kv1.4/Kvβ1.1 and Kv1.1/Kvβ1.1 might have the potential to reduce the severity of epileptic seizures. Moreover, it was shown that openers of Kv1.4 channels, such as riluzole for the treatment of the degenerative motor neuron disease amyotrophic lateral sclerosis, might serve as neuroprotective agents^30^,^31^. Thus, in the above cases, Ca^2+^/CaM-mediated slow inactivation coupled to Kv1.1 and Kv1.4 channels might serve as an endogenous neuroprotective mechanism. A similar mechanism is anticipated for Kv1.1 and Kv1.4 channels in dorsal root ganglia neurons^32^, where Ca^2+^/CaM likely controls the activity of Kv1.1/Kvβ1.1 and Kv1.4/Kvβ1.1 channels with the effect to limit DRG neuronal output and, hence, contribute to control pain signaling. Overall, the strongest regulatory influence is expected in axons or nerve terminals where the expression of Kvβ1.1 subunits colocalizes with sites of Ca^2+^ entry into the cytosol.

Here we showed that elevated intracellular Ca^2+^ concentrations slow down inactivation induced by Kvβ1.1 in Kv1.1, as well as in Kv1.4 channels, but also that this effect requires the Ca^2+^-binding protein calmodulin. This result appears to be in contrast to an earlier report^23^ according to which Ca^2+^ alone can decelerate Kvβ1.1-mediated inactivation. Although we cannot offer an unequivocal explanation for the results by Jow *et al.* (2004)^23^, we demonstrated that 1 μM free Ca^2+^ applied to the cytosolic face of clean membrane patches of *Xenopus* oocytes does not eliminate Kvβ1.1-mediated inactivation, while a combination of CaM and Ca^2+^ (1 μM each) does (Fig. 3, Supplementary Fig. 2). Furthermore, there is a clear hit for a potential CaM-binding motif in the N-terminal structure of Kvβ1.1 (between position 32 and 56, Fig. 1a), and mutations in this domain render the Kvβ1.1 subunits insensitive towards intracellular Ca^2+^/CaM. Finally, recombinant CaM physically interacts with Kvβ1.1 subunits, but not with mutants with impaired CaM-binding motifs (Fig. 3f). Thus, Kvβ1.1 subunits gain their Ca^2+^ dependence from association with CaM, similar to other ion channels, such as cyclic nucleotide-gated channels^33^, N-methyl-D-aspartate receptors^34^, Ca^2+^-activated K^+^ channels of intermediate and small conductance^35^, and EAG1 channels (Kv10.1)^36^, to name a few. In all such cases, the Ca^2+^ sensitivity of CaM couples the channels to the intracellular Ca^2+^ concentration in a physiological range of a few 100 nM, thus enabling the channels to quickly respond to moderate excursions from resting [Ca^2+^]_i_ levels. Unlike to Ca^2+^-activated K^+^ channels of intermediate and small conductance^35^, however, CaM only undergoes a loose interaction with Kvβ1.1 subunits because CaM only binds to the Kvβ1.1 protein in the presence of free Ca^2+^ (Fig. 3f), and prebound CaM can be washed off excised membrane patches in the absence of Ca^2+^ (Supplementary Fig. 2).

Binding of calmodulin to the flexible “chain” region of Kvβ1.1 must be expected to restrain the mobility of the “ball” and, hence, to interfere with channel inactivation. Restraining of the N terminus has also been proposed as mechanism underlying the redox modulation of Kvβ1.1^22^. Oxidation of Kvβ-bound NADPH induces a conformational rearrangement of the Kvβ core domain allowing the interaction of negative charges (E265, E349) on the surface of the core with positive residues in the chain (R37, R48), thereby leading to slower inactivation kinetics^22^. Based on this mechanism, it is conceivable to assume that Ca^2+^/CaM interacts with the Kvβ core domain and causes ball immobilization by binding of the chain to the core domain residues E265 and E349. In this case, redox regulation and Ca^2+^/CaM regulation would share exactly the same mechanism to interfere with inactivation. This hypothesis was disproven by our results of the Kvβ1.1 Ca^2+^ sensitivity in the background of an E349K mutation in the core domain. In this setting, the redox regulation was abolished, while Ca^2+^ regulation of inactivation was fully conserved. Thus, various regulatory mechanisms converge on the Kvβ1.1 channel subunit to fine-tune the kinetics of Kv channels in response to physiological parameters such as [Ca^2+^]_i_, redox potential, and phosphorylation signaling. While all of these regulation events ultimately lead to diminished “ball” mobility and slower inactivation via Kvβ1.1, the precise molecular mechanisms and the amino acids involved are specific for each regulatory pathway. The exact knowledge of these modulation mechanisms is an important precondition for efforts to establish Kvβ1.1 as a target for a pharmacologic control of Kv1 channels.

## Methods

### Channel constructs and mutagenesis

In this study we used the following constructs: Kv channels subunits rat Kv1.1 (rKv1.1, KCNA1) and rat Kv1.4 (rKv1.4, KCNA4); (b) Kvβ subunit human Kvβ1.1 (hKvβ1.1, KCNB1 isoform 1). An overlap-extension mutagenesis approach as described previously^37^ was used to generate the following point mutants of hKvβ1.1: C7S, R37N-R41N-R48N (Kvβ1.1-RRR), F40S-F53S (Kvβ1.1-FF), and E349K.

### GST fusion proteins and pull-down assay

Full-length cDNA of human Kvβ1.1 and point mutants thereof (RRR, FF) were cloned into the pGEX-5X vector (GE Healthcare) to encode GST fusion proteins. The recombinant fusion proteins were expressed in *E. coli* BL21 cells and purified using glutathione sepharose 4 fast flow (GE Healthcare). Recombinant human calmodulin (CaM) was purified as described previously^36^. Immobilized GST fusion proteins were washed on the GSH beads with either Ca^2+^-free buffer (PBS-EGTA), or with Ca^2+^-containing buffer (PBS-Ca). Each 20 μl beads were mixed with 20 μl purified CaM (1 μg/μl) and 400 μl PBS-EGTA or PBS-Ca, respectively, and incubated with shaking for 1 h at 4 °C. The beads were centrifuged for 1 min at 4000 g and washed three times with the respective buffer. Subsequently, the resin-bound proteins were eluted with SDS-PAGE loading buffer, separated on 12% acrylamide gels and stained with Coomassie blue. The PBS buffers (pH 7.4) contained (in mM): 137 NaCl, 2.7 KCl, 10 Na_2_HPO_4_, 1.8 KH_2_PO_4_, plus 10 EGTA (PBS-EGTA) or 2 CaCl_2_ (PBS-Ca), respectively.

### *In-silico* search for potential calmodulin binding sites

The N-terminal sequences of various Kvβ subunits were subjected to an analysis searching for potential binding sites for calmodulin according to Mruk *et al.* (2014)^25^.

### Channel expression in *Xenopus* oocytes and HEK 293T cells

Capped mRNA was synthesized *in vitro* using the mMessage mMachine kit (Ambion, Austin, TX, USA). Oocytes were surgically removed from the ovarian tissue of *Xenopus laevis* that had been anesthetized by immersion in ice water⁄tricaine according to the local animal care program. The oocytes were defolliculated, and healthy stage V and VI oocytes were isolated and microinjected with 50 nl of a solution containing channel wild-type or mutant mRNA. In co-expression experiments, the ratio of mRNA coding for α and β subunits was 1:3. Electrophysiological measurements were performed 2–4 days after mRNA injection.

HEK 293T cells were transiently transfected using the Roti-Fect transfection kit (Carl Roth, Karlsruhe, Germany). Dynabeads (Deutsche Dynal GmbH, Hamburg, Germany) were used for visual identification of individual cells, cotransfected with CD8. The ratio of DNA coding for α and β subunits was 1:3. Electrophysiological recordings were performed 2–3 days after transfection.

### Electrophysiological measurements

Ionic currents were recorded using the inside-out or whole-cell configuration at room temperature using an EPC-9 patch-clamp amplifier operated with PatchMaster software (both HEKA Elektronik, Lambrecht, Germany). Inside-out patch-clamp experiments were carried out with *Xenopus* oocytes; macroscopic currents were measured using aluminum silicate glass pipettes with resistances of about 1 MΩ. The intracellular solutions contained (in mM): “0 Ca^2+^”, 100 K-aspartate, 15 KCl, 10 EGTA, 10 4-(2-hydroxyethyl)-1-piperazineethanesulfonic acid (HEPES), or “1 μM Ca^2+^”, 100 K-aspartate, 15 KCl, 5.4 CaCl_2_, 10 HEDTA, 10 HEPES, both (pH 8.0 with KOH). The extracellular solution contained (in mM): 103.6 Na-aspartate, 11.4 KCl, 1.8 CaCl_2_, 10 HEPES (pH 7.2 with NaOH). Free Ca^2+^ concentrations were estimated with WEBMAXC software ( http://maxchelator.stanford.edu). Solution changes in patch-clamp experiments were performed using a multi-channel perfusion system in which the patch was placed directly in the center of streaming solution. Calmodulin was applied to inside-out patches with the intracellular solution (either 0 or 1 μM free Ca^2+^).

Whole-cell voltage-clamp experiments were carried out with transiently transfected HEK 293T cells. Patch pipettes from borosilicate glass with resistances of 0.9–2.0 MΩ were used. The pipette solution contained (in mM): “0 Ca^2+^”,140 KCl, 10 EGTA, 10 HEPES (pH 7.3 with KOH); or “1 μM Ca^2+^”,140 KCl, 10 HEDTA, 1.7 CaCl_2,_ 10 HEPES (pH 7.3 with KOH); bath solution (in mM): 146 NaCl, 4 KCl, 2 MgCl_2_, 2 CaCl_2_, 10 HEPES (pH 7.4 with NaOH). To study the effect of intracellular Ca^2+^ in the whole-cell patch-clamp mode, the intracellular solution was buffered with 100 μM EGTA to remove cell free Ca^2+^, and ionomycin (1 μM, Sigma-Aldrich) was applied via the bath solution to elevate intracellular [Ca^2+^]. Cells with series resistance above 5 MΩ were discarded; the series resistance was compensated electronically for by more than 70%. Leak and capacitance currents were subtracted using a p/6 correction method.

### Data Analysis

Data were analyzed with FitMaster (HEKA Elektronik) and IgorPro (WaveMetrics, Lake Oswego, OR, USA). Averaged data are presented as means ± s.e.m. (n = number of independent measurements) unless specified otherwise. Averaged data were compared with a two-sided Student’s t-test or Wilcoxon signed rank test as applicable. The resulting *P* values are specified.

## Additional Information

**How to cite this article**: Swain, S. M. *et al.* Ca^2+^/calmodulin regulates Kvb1.1-mediated inactivation of voltage-gated K^+^ channels. *Sci. Rep.* **5**, 15509; doi: 10.1038/srep15509 (2015).

## Supplementary Material

Supplementary Information

## References

[b1] Lipton S. A. & Rosenberg P. A. Excitatory amino acids as a final common pathway for neurologic disorders. N. Engl. J. Med. 330, 613–622 (1994). 7905600 10.1056/NEJM199403033300907

[b2] Giese K. P. *et al.* Reduced K^+^ channel inactivation, spike broadening, and after-hyperpolarization in Kvβ1.1-deficient mice with impaired learning. Learn. Mem. 5, 257–273 (1998). 10454353 PMC311244

[b3] Hoshi T., Zagotta W. N. & Aldrich R. W. Biophysical and molecular mechanisms of Shaker potassium channel inactivation. Science 250, 533–538 (1990). 2122519 10.1126/science.2122519

[b4] Zagotta W. N., Hoshi T. & Aldrich R. W. Restoration of inactivation in mutants of Shaker potassium channel peptide derived from ShB. Science 250, 568–571 (1990). 2122520 10.1126/science.2122520

[b5] Antz C. *et al.* Control of K^+^ channel gating by protein phosphorylation: structural switches of the inactivation gate. Nat. Struct. Biol. 6, 146–150 (1999). 10048926 10.1038/5833

[b6] Ruppersberg J. P. *et al.* Regulation of fast inactivation of cloned mammalian I_K_(A) channels by cysteine oxidation. Nature 352, 711–714 (1991). 1908562 10.1038/352711a0

[b7] Rettig J. *et al.* Inactivation properties of voltage-gated K^+^ channels altered by presence of β-subunit. Nature 369, 289–294 (1994). 8183366 10.1038/369289a0

[b8] Heinemann S. H., Rettig J., Graack H.-R. & Pongs O. Functional characterization of Kv channel β-subunits from rat brain. J. Physiol. 493, 625–633 (1996). 8799886 10.1113/jphysiol.1996.sp021409 PMC1159012

[b9] Pongs O. & Schwarz J. R. Ancillary subunits associated with voltage-dependent K^+^ channels. Physiol. Rev. 90, 755–796 (2010). 20393197 10.1152/physrev.00020.2009

[b10] McCormack T. & McCormack K. Shaker K^+^ channel β subunits belong to an NAD(P)H-dependent oxidoreductase superfamily. Cell 79, 1133–1135 (1994). 8001150 10.1016/0092-8674(94)90004-3

[b11] Gulbis J. M., Zhou M., Mann S. & MacKinnon R. Structure oft he cytoplasmic β subunit–T1 assembly of voltage-dependent K^+^ channels. Science 289, 123–127 (2000). 10884227 10.1126/science.289.5476.123

[b12] Long S. B., Campbell E. B. & MacKinnon R. Crystal structure of a mammalian voltage-dependent *Shaker* family K^+^ channel. Science 309, 897–903 (2005). 16002581 10.1126/science.1116269

[b13] Stephens G. J. *et al.* Studies on the blocking action of human Kv3.4 inactivation peptide variants in the mouse cloned Kv1.1 K^+^ channel. J. Physiol. 496, 145–154 (1996). 8910203 10.1113/jphysiol.1996.sp021672 PMC1160831

[b14] Sahoo N., Hoshi T. & Heinemann S. H. Oxidative modulation of voltage-gated potassium channels. Antioxid. Redox Sig. 21, 933–952 (2014). 10.1089/ars.2013.5614 PMC4116129 24040918

[b15] Covarrubias M., Wei A., Salkoff L. & Vyas T. B. Elimination of rapid potassium channel inactivation by phosphorylation of the inactivation gate. Neuron 13, 1403–1412 (1994). 7993631 10.1016/0896-6273(94)90425-1 PMC2211371

[b16] Roeper H., Lorra C. & Pongs O. Frequency-dependent inactivation of mammalian A-type K^+^ channel Kv1.4 regulated by Ca^2+^/calmodulin-dependent protein kinase. J. Neurosci. 17, 3379–3391 (1997). 9133364 10.1523/JNEUROSCI.17-10-03379.1997 PMC6573714

[b17] Kwak Y. G. *et al.* Protein kinase A phosphorylation alters Kvβ1.3 subunit-mediated inactivation of the Kv1.5 potassium channel. J. Biol. Chem. 274, 13928–13932 (1999). 10318802 10.1074/jbc.274.20.13928

[b18] Oliver D. *et al.* Functional conversion between A-type and delayed rectifier K^+^ channels by membrane lipids. Science 304, 265–270 (2004). 15031437 10.1126/science.1094113

[b19] Padanilam B. J. *et al.* Molecular determinants of intracellular pH modulation of human Kv1.4 N-type inactivation. Mol. Pharmacol. 62, 127–134 (2002). 12065763 10.1124/mol.62.1.127

[b20] Weng J., Cao Y., Moss N. & Zhou M. Modulation of voltage-dependent Shaker family potassium channels by an aldo-keto reductase. J. Biol. Chem. 281, 15194–15200 (2006). 16569641 10.1074/jbc.M513809200 PMC2862575

[b21] Pan Y., Weng J., Cao Y., Bhosle R. C. & Zhou M. Functional coupling between the Kv1.1 channels and aldoketoreductase Kvβ1. J. Biol. Chem. 283, 8634–8642 (2008). 18222921 10.1074/jbc.M709304200 PMC2417172

[b22] Pan Y., Weng J., Levin E. J. & Zhou M. Oxidation of NADPH on Kvβ1 inhibits ball-and-chain type inactivation by restraining the chain. PNAS 108, 5885–5890 (2011). 21436029 10.1073/pnas.1100316108 PMC3078402

[b23] Jow F., Zhang Z. H., Kopsco D. C., Carroll K. C. & Wang K. Functional coupling of intracellular calcium and inactivation of voltage-gated Kv1.1/Kvβ1.1 A-type K^+^ channels. PNAS 101, 15535–15540 (2004). 15486093 10.1073/pnas.0402081101 PMC524431

[b24] Decher N. *et al.* Structural determinants of Kvβ1.3 induced channel inactivation: a hairpin modulated by PIP_2_. EMBO J. 27, 3164–3174 (2008). 18987637 10.1038/emboj.2008.231 PMC2599874

[b25] Mruk K., Farley B. M., Ritacco A. W. & Kobertz W. R. Calmodulation meta-analysis: predicting calmodulin binding via canonical motif clustering. J. Gen. Physiol. 144, 105–114 (2014). 24935744 10.1085/jgp.201311140 PMC4076516

[b26] Rhodes K. J. *et al.* Association and colocalization of the Kvβ1 and Kvβ2 β-subunits with Kv1 α-subunits in mammalian brain K^+^ channel complexes. J. Neurosc. 17, 8246–8258 (1997). 10.1523/JNEUROSCI.17-21-08246.1997 PMC6573739 9334400

[b27] Geiger J. R. P. & Jonas P. Dynamic control of presynaptic Ca^2+^ inflow by fast-inactivating K^+^ channels in hippocampal mossy fiber boutons. Neuron 28, 927–939 (2000). 11163277 10.1016/s0896-6273(00)00164-1

[b28] Debanne D., Campanac E., Bialowas A., Carlier E. & Alcaraz G. Axon physiology. Physiol. Rev. 91, 555–602 (2011). 21527732 10.1152/physrev.00048.2009

[b29] Schulte U. *et al.* The epilepsy-linked Lgi1 protein assembles into presynaptic Kv1 channels and inhibits inactivation by Kvβ1. Neuron 49, 697–706 (2006). 16504945 10.1016/j.neuron.2006.01.033

[b30] Xu L., Enyeart J. A. & Enyeart J. J. Neuroprotective agent riluzole dramatically slows inactivation of Kv1.4 potassium channels by a voltage-dependent oxidative mechanism. J. Pharmacol. Exp. Ther. 299, 227–237 (2001). 11561084

[b31] Takeda M. *et al.* Potassium channels as a potential therapeutic target for trigeminal neuropathic and inflammatory pain. Mol. Pain 7, 5 (2011). 21219657 10.1186/1744-8069-7-5 PMC3024960

[b32] Binzen U. *et al.* Co-expression of the voltage-gated potassium channel Kv1.4 with transient receptor potential channels (TRPV1 and TRPV2) and the cannabinoid receptor CB1 in rat dorsal root ganglion neurons. Neurosc. 142, 527–539 (2006). 10.1016/j.neuroscience.2006.06.020 16889902

[b33] Hsu Y. T. & Molday R. S. Modulation of the cGMP-gated channel of rod photoreceptor cells by calmodulin. Nature 361, 76–79 (1993). 7678445 10.1038/361076a0

[b34] Ehlers M. D., Zhang S., Bernhardt J. P. & Huganir R. L. Inactivation of NMDA receptors by direct interaction of calmodulin with the NR1 subunit. Cell 84, 745–755 (1996). 8625412 10.1016/s0092-8674(00)81052-1

[b35] Xia X.-M. *et al.* Mechanism of calcium gating in small-conductance calcium-activated potassium channels. Nature 395, 503–507 (1998). 9774106 10.1038/26758

[b36] Ziechner U. *et al.* Inhibition of human *ether à go-go* potassium channels by Ca^2+^/calmodulin binding to the cytosolic N- and C-termini. FEBS J. 273, 1074–1086 (2006). 16478480 10.1111/j.1742-4658.2006.05134.x

[b37] Schönherr R., Löber K. & Heinemann S. H. Inhibition of human *ether à go-go* potassium channels by Ca^2+^/calmodulin. EMBO J. 19, 3263–3271 (2000). 10880439 10.1093/emboj/19.13.3263 PMC313935

