# Supplementary material for: Ca2+/calmodulin regulates Kvβ1.1-mediated inactivation of voltage-gated K+ channels
Source: Sci Rep. 2015 Oct 21;5:15509. doi: 10.1038/srep15509 (PMC4614385; doi:10.1038/srep15509)
Supplement: Supplementary Information [file srep15509-s1.pdf]

Ca^{2+}/calmodulin regulates Kvβ1.1-mediated inactivation of voltage-gated K^+ channels

Sandip M. Swain[1], Nirakar Sahoo[1,2], Sophie Dennhardt[1], Roland Schönherr[1], and Stefan H. Heinemann[1]

[1]Center for Molecular Biomedicine, Department of Biophysics, Friedrich Schiller University Jena & Jena University Hospital, Hans-Knöll-Str. 2, D-07745 Jena, Germany,

[2]Present address: University of Michigan, Department of MCDB, 830 North University Avenue, Ann Arbor, MI 48109-1048, USA

Corresponding author:

Prof. Dr. Stefan H. Heinemann
Center for Molecular Biomedicine
Department of Biophysics
Friedrich Schiller University Jena & Jena University Hospital
Hans-Knöll-Str. 2
D-07745 Jena, Germany
Tel: ++49-3641-9 39 56 50
Fax: ++49-3641-9 39 56 52
E-Mail: Stefan.H.Heinemann@uni-jena.de

Running title: Ca^{2+} dependence of Kvβ1.1

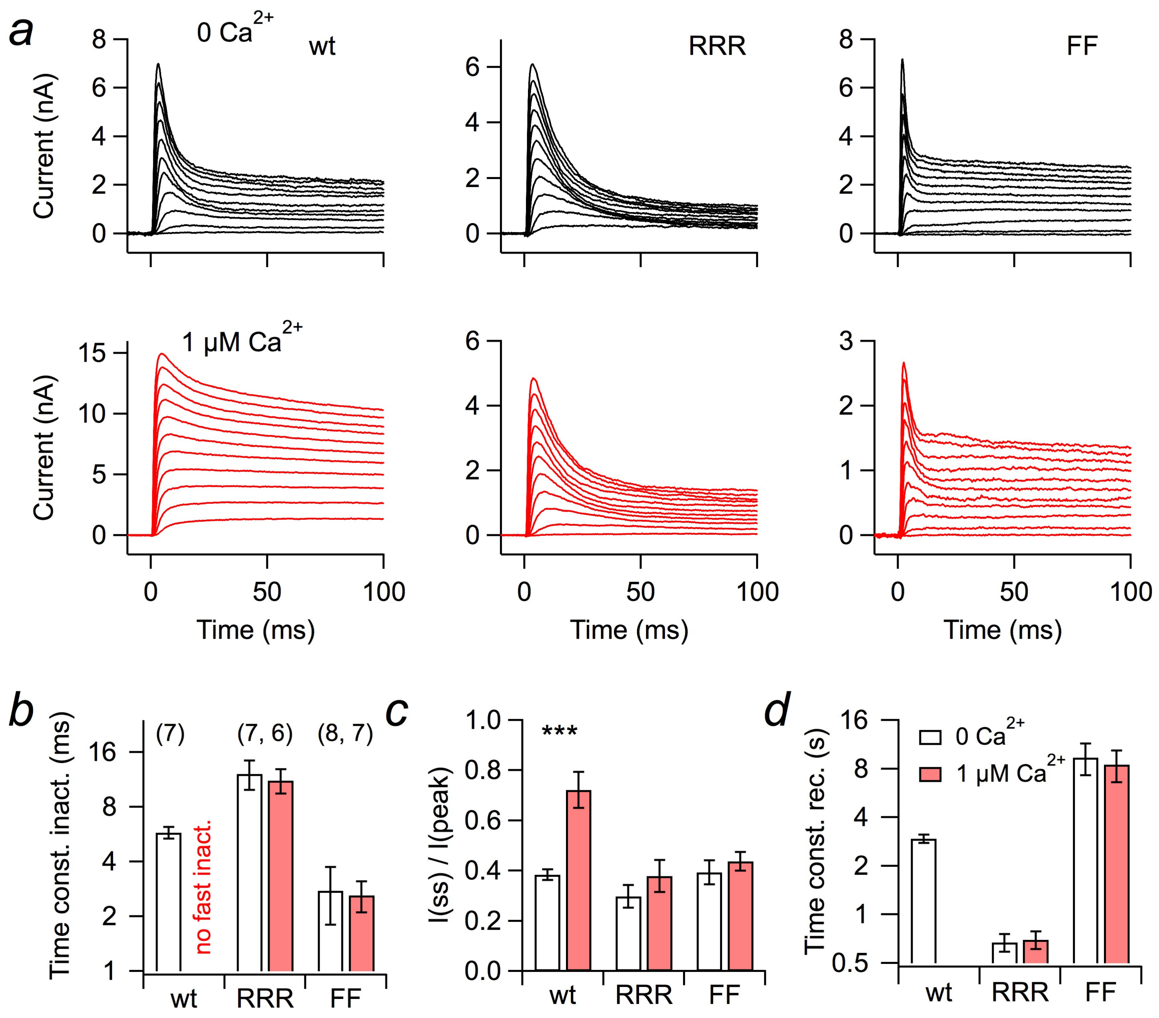

Supplementary Figure 1 | Whole-cell recordings of Kv1.1 currents from HEK 293T cells. Kv1.1 channels were coexpressed with Kvβ1.1 wild type (wt) or mutants RRR and FF in HEK 293T cells; currents were measured in the whole-cell mode. (a) Current traces upon depolarization to –40 through 60 mV in steps of 10 mV from a holding voltage of –90 mV for the indicated Kvβ1.1 constructs. For the top traces (black), the pipette solution contained 10 mM EGTA, thus the concentration of free Ca^{2+} was negligible; for the bottom traces (red), free [Ca^{2+}] was adjusted to 1 µM (in mM: 140 KCl, 1.7 $CaCl_2$, 10 HEDTA, 10 HEPES, pH 7.3 with KOH). (b) Time constant of rapid inactivation at 50 mV without (white) and with Ca^{2+} (red). (c) Ratio of remaining current after 100 ms at 50 mV and peak current. (d) Time constant of recovery from inactivation at –90 mV. Data in b-d are mean ± s.e.m. with n indicated in parentheses of panel b. Two-sided t-test: *** $P < 0.001$.

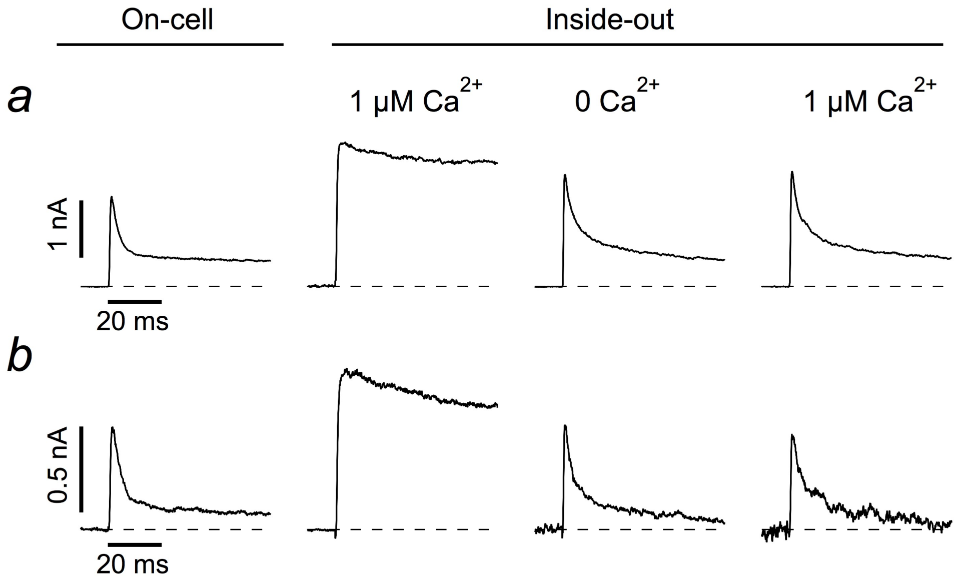

Supplementary Figure 2 | Acutely excised patches retain some CaM to facilitate Ca^{2+}-induced loss of Kvβ1.1-mediated inactivation. (a, b) Two examples of current traces elicited with depolarizing steps to 50 mV of Kv1.1+Kvβ1.1 complexes expressed in *Xenopus* oocytes: on-cell mode followed by the inside-out configuration into bath solutions with the indicated concentration of free Ca^{2+} and no extra calmodulin.
